# Exploring the Impact of Mitonuclear Discordance on Disease in Latin American Admixed Populations

**DOI:** 10.3390/genes16060638

**Published:** 2025-05-27

**Authors:** Mauricio Ruiz, Daniela Böhme, Gabriela M. Repetto, Boris Rebolledo-Jaramillo

**Affiliations:** Rare Diseases Program, Center for Genetics and Genomics, Institute of Science and Innovation in Medicine, Facultad de Medicina, Clínica Alemana Universidad del Desarrollo, Santiago 7610615, Chile; ruiz.moraga.90@gmail.com (M.R.); danielabohme@udd.cl (D.B.); grepetto@udd.cl (G.M.R.)

**Keywords:** mitonuclear discordance, admixed Latinos, mitochondrial genomics

## Abstract

**Background**. The coevolution of nuclear and mitochondrial genomes has guaranteed mitochondrial function for millions of years. The introduction of European (EUR) and African (AFR) genomes into the Ameridian continent during the Columbus exchange in Latin America created an opportunity to naturally test different combinations of nuclear and mitochondrial genomes. However, the impact of potential “mitonuclear discordance” (MND, differences in ancestries) has not been evaluated in Latin American admixed individuals (AMR) affected with developmental disorders, even though MND alters mitochondrial function and reduces viability in other organisms. **Methods**. To characterize MND in healthy and affected AMR individuals, we used AMR genotype data from the 1000 Genomes Project (n = 385), two cohorts of 22q.11 deletion syndrome patients 22qDS-ARG (n = 26) and 22qDS-CHL (n = 58), and a cohort of patients with multiple congenital anomalies and/or neurodevelopmental disorders (DECIPHERD, n = 170). Based on their importance to mitochondrial function, genes were divided into all mitonuclear genes (n = 1035), high-mt (n = 167), low-mt (n = 793), or OXPHOS (n = 169). We calculated local ancestry using FLARE and estimated MND as the fraction of nuclear mitochondrial genes ancestry not matching the mtDNA ancestry and ∆MND as (MNDoffspring—MNDmother)/MNDmother. **Results.** Generally, MND showed distinctive population and haplogroup distributions (ANOVA *p* < 0.05), with haplogroup D showing the lowest MND of 0.49 ± 0.17 (mean ± s.d.). MND was significantly lower in 22qDS-ARG patients at 0.43 ± 0.24 and DECIPHERD patients at 0.56 ± 0.12 compared to healthy individuals at 0.60 ± 0.09 (ANOVA *p* < 0.05). OXPHOS and high-mt showed the same trend, but with greater differences between healthy and affected individuals. **Conclusions**. MND seems to inform population history and constraint among affected individuals, especially for OXPHOS and high-mt genes.

## 1. Introduction

### 1.1. Mitochondrial Function Depends on Nuclear and Mitochondrial Factors

Mitochondria are double-layered organelles critical for energy production and other essential functions such as metabolism, apoptosis, and immune responses [1,2,3,4,5]. They have their own genome: the mitochondrial DNA (mtDNA) [6,7], but the majority of mitochondrial proteins (~1000) are encoded by nuclear genes [8,9,10]. Consequently, the assembly of mitochondrial respiratory complexes depends on tightly coordinated nuclear and mitochondrial gene expression, maintained through mitonuclear coevolution [11].

### 1.2. Mitonuclear Discordance Leads to Dysfunctional Mitochondria

Studies in model organisms show that mitonuclear discordance (MND), a mismatch between nuclear and mitochondrial ancestries, leads to mitonuclear incompatibility, reducing organismal fitness via disrupted oxidative phosphorylation (OXPHOS) [12,13,14,15]. These effects are often reversed through maternal backcrossing [16] and have influenced species divergence [17,18], underscoring the importance of mitonuclear compatibility in evolutionary fitness.

### 1.3. Admixture Leads to Mitonuclear Discordance in Humans

In humans, admixture between historically isolated populations can generate MND [19,20]. The high mutation rate of mtDNA [21] led to diverse haplogroups, with A, B, C, and D populating the Americas around 20,000 years ago [22]. Following the Columbus exchange, African, European, and Native American ancestries began to mix [23,24], producing novel mitonuclear combinations now common in Latin Americans [25]—yet their health implications remain understudied.

### 1.4. The Effect of Mitonuclear Discordance Due to Admixture Is Unclear

Concerns about mitonuclear incompatibility emerged with mitochondrial replacement therapy (MRT), which combines nuclear and mitochondrial genomes from different individuals [26]. While some studies found no adverse effects from MND in healthy adults [19,27], others showed that increased MND correlates with lower mtDNA copy number—a potential disease biomarker [20,28,29,30]. Recent analyses using mother–offspring pairs suggest selective pressures may favor mitonuclear compatibility, indicating that MND could impact early development [31,32].

### 1.5. Mitonuclear Interactions Can Modulate Disease Phenotypes

Recent studies show that mitonuclear interactions can shape disease risk. For example, mtDNA haplogroups enhance polygenic risk prediction in neurodegenerative disorders [33,34,35], and disruptions in nuclear–mitochondrial protein interactions are linked to diabetes [36]. Given the dual genetic control of mitochondrial function, both mtDNA variation and MND may act as genetic modifiers [37], contributing to phenotypic variability in developmental disorders such as 22q11.2 deletion syndrome, which involves the loss of six nuclear-encoded mitochondrial genes [38,39,40,41,42]. Haploinsufficiency of *MRPL40*, one such gene, impairs mitochondrial function [43] and may exacerbate disease in the context of high MND. Thus, we hypothesize that MND contributes to disease and assess its role by comparing MND in healthy and affected admixed individuals.

## 2. Materials and Methods

### 2.1. Datasets

We reused data from multiple sequencing projects. We analyzed the genome of 385 samples of Latin American admixed ancestry from the 1000 Genome Project Phase 3 dataset (1kGP-AMR) [44], which included 98 mother–offspring pairs (1kGP-pairs); the exome of 172 Chilean samples from the Decoding Complex Inherited Phenotypes in Rare Disorders cohort (DECIPHERD, referred to as DRD for short) [45], a cohort where probands are affected by unknown rare disorders, causing congenital anomalies and/or neurodevelopmental disorders, which included 75 proband-only (DRD-affected) and 32 trios, from which healthy parents were used as controls (DRD-healthy) and mothers with offspring as pairs (DRD-pairs); the exome of 58 Chilean 22q11.2 deletion syndrome (22qDS) patients (22q-CHL); and the exome of 26 Argentinian 22qDS patients (22q-ARG), which included 13 mother–offspring pairs (22q-ARG-pairs) [46] (see Table 1).

### 2.2. Imputation of Exome Data

Exome data were imputed with the Michigan Imputation Server 2 (https://imputationserver.sph.umich.edu (accessed on 25 September 2024)) [47], using the reference panel “1000G Phase 3 30x (GRCh38/hg38)”, and filtered using rsq = 0.3.

### 2.3. Ancestry Inference

We calculated global ancestry with ADMIXTURE v.1.3 [48] using k = 5 to determine which 1kGP samples (out of 3202 samples) would serve as Native American (NAT), European (EUR), and African (AFR) population references (admixture ≤ 1% for EUR and AFR, ≤10% for admixed American (AMR)). We chose k = 5 because the diversity in the 1kGP is represented by five source populations: EUR, AFR, AMR, South Asian (SAS), and East Asian (EAS) [27]. The 1kGP dataset included 63,993,320 single-nucleotide variants (SNVs), but we focused on 14,804,207 SNVs known to indicate Latin American admixed ancestry according to gnomAD v.3 [49]. Then, for each dataset, we calculated local ancestry using FLARE v.0.5.1 [50] using three source populations: NAT, AFR, EUR, and default parameters. Global ancestry estimations from local ancestry averages can be found in Figure S1.

### 2.4. Haplogroup Assignment

MtDNA haplogroups were calculated using the web version of Haplogrep (https://haplogrep.i-med.ac.at/), PhyloTree Build 17 [51] on the off-target mtDNA reads obtained from exome sequencing and processed with the GATK Best Practices for mitochondrial short variant discovery protocol (https://gatk.broadinstitute.org/hc/en-us/articles/4403870837275-Mitochondrial-short-variant-discovery-SNVs-Indels (accessed on 25 September 2024)). Haplogroup assignment can be found in Table S1.

### 2.5. Mitonuclear Discordance

Mitonuclear discordance has been defined as the fraction of nuclear ancestry not matching the mtDNA ancestry [20]. For example, in an individual with global nuclear components that are 55% European, 40% Native American, and 5% African, with Native American mtDNA haplogroup B, MND would be 60% (the sum of European and African components). For this work, we calculated MND for each gene independently. Since each gene was represented by a set of SNPs of known local ancestry, we calculated the proportion of SNPs not matching the haplogroup’s ancestry and called that the “gene-wise MND”. We then averaged the gene-wise MND over all genes belonging to a particular gene set, e.g., “all mitonuclear genes” or “OXPHOS genes”, and compared different sample sets. By default, we show the results for all mitonuclear genes, unless otherwise noted. We also defined the intergenerational change in mitonuclear discordance as: ∆MND = (MND_offspring_ − MND_mother_)/MND_mother_.

### 2.6. Gene Sets

Nuclear-encoded mitochondrial genes were classified into “all mitonuclear” (n = 1035), “OXPHOS” (n = 169), according to MitoCarta v.3.0 [8], and “high-mt” (n = 167) or “low-mt” (n = 793), according to Sloan et al. (2015) [52].

### 2.7. Statistics

Results are shown as mean ± standard deviation, unless otherwise noted. Multiple group comparisons were calculated using ANOVA for all cases where normality or the central limit theorem was applicable, otherwise, we used Kruskal–Wallis. Pairwise comparisons were calculated with the *t*-test or Mann–Whitney U test, accordingly. Multiple hypotheses testing was controlled with Bonferroni’s correction. For all tests, the significance level alpha was set at 0.05. We used R v.4.3.3 to perform all statistical analyses and plots.

## 3. Results

### 3.1. Imputation

We leveraged existing datasets to describe MND and ∆MND in healthy and affected individuals. Out of the 3202 1000 Genomes Project Phase 3 individuals [44], 336 African, 198 European, and 25 admixed American individuals served as population references for all calculations. After imputation of exome data, we obtained 6,279,408 22q-ARG SNVs; 10,642,230 22q-CHL SNVs; 2,361,135 DRD-pairs and DRD-healthy SNVs; and 8,504,595 DRD-affected SNVs. We categorized the cohorts according to Table 1.

### 3.2. MND in Healthy Individuals Shows Strong Population and Haplogroup-Specific Distributions

**Figure 1:**
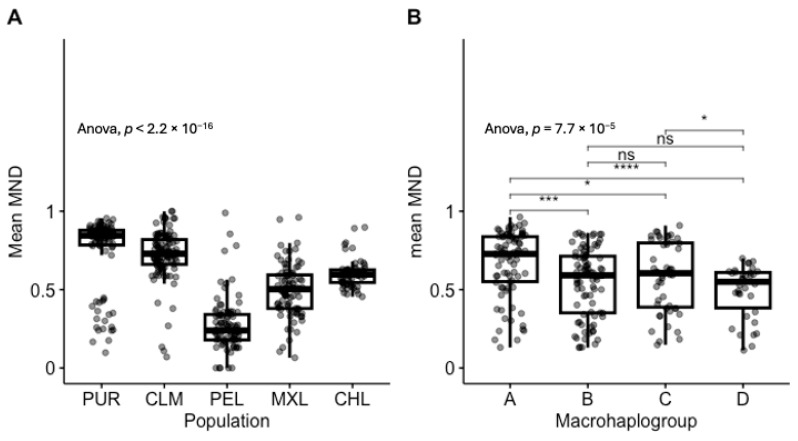
Population- and haplogroup-specific MDN distribution. (**A**) Distribution of mean MND among 1kGP and Chilean populations. (**B**) Distribution of mean MND among Latin American macrohaplogroups. ****: *p* < 0.0001: ***: *p* < 0.001; *: *p* < 0.05; ns: not significant.

First, we calculated mean MND distributions for all populations and observed strong population specificity: Puerto Rican (PUR, 0.75 ± 0.22); Colombian (CLM, 0.72 ± 0.16); Peruvian (PEL, 0.27 ± 0.18); Mexican (MXL, 0.49 ± 0.18); Chilean, (CHL 0.60 ± 0.09); ANOVA F = 112.7, *p* = 3.37 × 10^−66^ (all pairwise comparisons were statistically significant, Figure 1A), and the mean of the distributions positively correlated with European ancestry, rho = 0.689, *p* = 2.42 × 10^−64^, Figure S2.

Then, we merged all Latin American haplogroups belonging to the same branch into single-letter macrohaplogroups to increase sample size and focused on individuals with haplogroups A–D only. We observed that macrohaplogroup A had the highest mean MND (0.67 ± 0.21), significantly different compared to macrohaplogroups B (0.55 ± 0.22), C (0.58 ± 0.22), and D (0.49 ± 0.17), which in turn had similar distributions, ANOVA F = 7.529 *p* = 7.7 × 10^−5^. Noticeably, macrohaplogroup D had the lowest mean MND and variance. Pairwise comparisons are shown in Figure 1B.

### 3.3. Affected Individuals Have Lower MND

**Figure 2:**
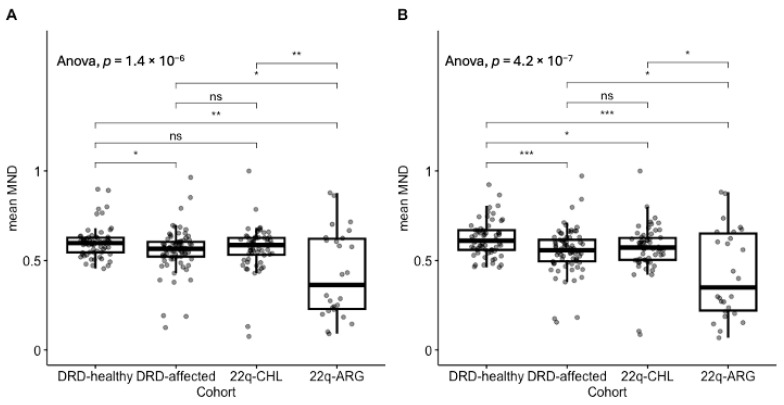
Mean MND in cohorts of patients with different genetic disorders. We compared healthy individuals to multiple cohorts of patients. The first three distributions correspond to Chilean individuals, whereas the last distribution corresponds to patients from Argentina. (**A**) Comparison of mean MDN using all mitonuclear genes. (**B**) Comparison of mean MDN using OXPHOS genes. DRD: DECIPHERD. CHL: Chile. ARG: Argentina. ***: *p* < 0.001; **: *p* < 0.01; *: *p* < 0.05; ns: not significant.

We compared the mean MND of healthy parents from the DECIPHERD cohort to the mean MND of affected cohorts. We observed that, in general, using all mitonuclear genes, MND in affected individuals was lower: DRD-healthy (0.60 ± 0.09); DRD-affected (0.56 ± 0.12); 22q-CHL (0.57 ± 0.13), with the 22q-ARG cohort showing the lowest mean MND (0.43 ± 0.24), ANOVA F = 10.7, *p* = 1.4 × 10^−6^. Interestingly, the variance of 22q-ARG patients was 3.6 times greater than the variance of their Chilean counterpart (0.058 and 0.016, respectively, *p* = 6.33 × 10^−5^) (Figure 2A). Similarly, we ran the same analyses, but focusing only on OXPHOS genes. We observed an even stronger difference between healthy and affected cohorts: healthy (0.62 ± 0.1), DRD-affected (0.55 ± 0.13), 22q-CHL (0.57 ± 0.13), and 22q-ARG (0.43 ± 0.25), ANOVA F = 11.6, *p* = 4.2 × 10^−7^ (Figure 2B and Table 2). The results for high-mt and low-mt genes are shown in Figure S3.

### 3.4. Signs of Constraint of ∆MND

**Figure 3:**
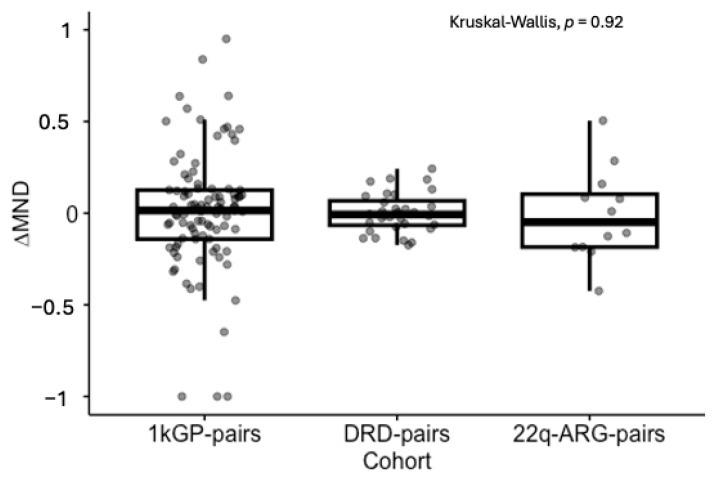
Comparison of ∆MND in affected cohorts. 1kGP-pairs represent healthy individuals, while DRD-pairs and 22q-ARG-pairs represent cohorts affected by rare genetic disorders. 1kGP: 1000 Genomes Project. DRD: DECIPHERD. ARG: Argentina.

We observed that the intergenerational change in MND had a lower median in DRD of −0.01 [−0.18–0.24] (median and range) and in 22q-ARG-pairs of −0.05 [−0.43–0.50], compared to that of healthy participants of 0.02 [−1.0–1.5], yet the distributions were not significantly different, Kruskal–Wallis *p* = 0.92. However, we observed significant differences in variance between healthy (s2 = 0.16) and DRD (s2 = 0.01) pairs, *p* = 7.0 × 10^−12^, but not between healthy and 22q-ARG-pairs (s2 = 0.06, *p* = 0.09), Figure 3.

## 4. Discussion

This study explored the contribution of mitonuclear discordance to disease in Latin American admixed populations, offering a novel perspective on the interplay between nuclear and mitochondrial genomes in diverse human cohorts. By leveraging comprehensive datasets, including the 1000 Genomes Project and cohorts of patients with rare genetic disorders, this research highlights the potential impact of MND on mitochondrial function and disease phenotypes.

Our results showed strong population- and haplogroup-specific distributions of MND, with significant correlations between MND and European ancestry, results that align with previous studies [20,53]. Notably, in our study, haplogroup D exhibited the lowest MND, suggesting potential evolutionary constraints. While direct studies on haplogroup D are limited, research has shown that certain mtDNA lineages may be subject to evolutionary constraints due to the interaction between haplogroup-defining variants. For example, in an H7A haplogroup background, the J haplogroup-defining variant m.13708G > A causes a complex phenotype including a combination of connective tissue, neurological, and metabolic symptoms, demonstrating that common nonpathogenic variation can cause mitochondrial dysfunction due to variant incompatibility [54,55].

The observed lower mitonuclear discordance in affected individuals compared to healthy controls, particularly pronounced in OXPHOS genes, suggests that MND may influence disease phenotypes. This underscores the critical role of OXPHOS in maintaining mitochondrial homeostasis, suggesting that higher MND levels could be detrimental to survival. Research has demonstrated that mitochondrial dysfunction, including impaired OXPHOS, is implicated in various diseases. For instance, mitochondrial dysfunction has been linked to the etiology of bipolar disorder, with evidence pointing to dysregulated OXPHOS and altered brain bioenergetics [56]. Additionally, studies have shown that mutations affecting OXPHOS can lead to severe disorders. For example, combined oxidative phosphorylation deficiency type 14, caused by mutations in the *FARS2* gene, manifests in conditions such as epileptic status and other neurological impairments [57]—which could explain some of the neurological phenotypes observed in the DECIPHERD cohort. These findings highlight the importance of mitonuclear interactions in disease manifestation and the necessity of coordinated function between nuclear and mitochondrial genomes for optimal OXPHOS activity. Disruptions in this coordination may compromise mitochondrial function and contribute to disease development.

We also examined intergenerational changes in MND, which revealed signs of constraint in affected cohorts. As expected, ∆MND in healthy individuals showed signs of genetic drift, since the distribution was centered around zero. While the median ∆MND was lower in affected mother–offspring pairs, these differences were not statistically significant, yet differences in variance were noted. This difference in variance may reflect selective pressures or mechanisms that limit mitonuclear discordance across generations. Studies have shown that mitonuclear interactions are subject to selective pressures, particularly in the context of disease. For example, research on admixed human populations has demonstrated that mitonuclear DNA discordance can affect mtDNA copy number or gene expression, with higher discordance leading to lower mtDNA copy numbers and lower gene expression. This suggests that mitonuclear compatibility is crucial for maintaining mitochondrial function and that selective pressures may act to minimize discordance across generations [20,53]. Additionally, studies on mitonuclear incompatibilities in allopatric speciation have highlighted that rapid evolution of the mitochondrial genome creates intrinsic selection pressures favoring nuclear gene mutations that maintain mitochondrial function. This indicates that mitonuclear interactions are under selective constraints to ensure compatibility, which may be particularly relevant in disease contexts where mitochondrial function is compromised [58].

Our results suggest that MND could act as a genetic modifier, contributing to phenotypic variability in diseases such as 22q11.2DS. This aligns with the growing recognition of mitonuclear interactions in modulating disease risk, especially in neurodegenerative, immunological, and developmental disorders. Our previous research demonstrated that mtDNA heteroplasmy may influence the incomplete penetrance of the palatal phenotype in 22q11.2DS, highlighting the potential role of mitochondrial variants within a specific genetic background as genetic modifiers [46]. Also, research on Alzheimer’s disease has indicated that mitonuclear interactions influence disease risk. Associations between mtDNA haplogroups and nuclear-encoded mitochondrial genes have been linked to variations in dementia risk and age of onset, underscoring the significance of mitonuclear interactions in neurodegenerative diseases [34].

One of our study’s strengths lies in its use of diverse datasets, allowing for robust comparisons across populations and disease states, and the focus on Latin American cohorts. Including diverse populations not only improves the generalizability of genetic findings but also ensures that the insights gained are relevant to groups historically excluded from genomic medicine. This is especially crucial for identifying ancestry-specific modifiers of disease risk and for promoting equity in biomedical research. While correlations were observed, mechanistic insights into how MND influences disease phenotypes require further investigation, particularly the exploration of functional consequences of MND in cellular and animal models, to hopefully develop clinical tools to assess MND as a potential biomarker for disease risk and progression.

One limitation of our study is the use of imputed exome data for local ancestry inference. Although imputation helps to increase variant density, the accuracy of inferred genotypes depends heavily on the quality of the reference panel and the degree of genetic similarity between the target and reference populations [59]. In admixed populations, where local ancestry can vary sharply across the genome, these limitations may lead to less precise ancestry estimates at mitochondrial-related nuclear loci. Consequently, the calculation of mitonuclear discordance may be affected by imputation biases, particularly in regions with low imputation quality, thus we chose a stringent filter to keep regions with high imputation quality (Materials and Methods). Future studies using whole-genome data and population-matched reference panels could improve the resolution and reliability of MND estimation.

## 5. Conclusions

We investigated the role of MND in disease among Latin American admixed populations, describing how mismatches between nuclear and mitochondrial ancestries could impact mitochondrial function and disease phenotypes. Utilizing datasets from the 1000 Genomes Project and cohorts with rare genetic disorders, we demonstrated population- and haplogroup-specific patterns of MND, with haplogroup D showing the lowest MND values. Affected individuals exhibited significantly lower MND than healthy controls, particularly in genes related to oxidative phosphorylation, suggesting potential links between MND and disease. Intergenerational analyses also revealed constraints on MND, pointing to selective pressures in disease contexts. These findings underscore the importance of mitonuclear interactions in shaping disease phenotypes and highlight the need for further research into their functional and clinical implications, particularly in underrepresented populations.

## Figures and Tables

**Table 1:** Datasets used for the calculation of baseline and disease-associated MND in Latin American individuals.

Dataset Origin	Short Name	# of Samples	# Pairs/ Trios	Usage
1000 Genomes Project Phase 3	1kGP-ALL	3202	602	To determine AMR, EUR, and AFR haplotype references
1000 Genomes Project Phase 3	1kGP-AMR	385	98	To describe MND and ∆MND in healthy Latin American individuals
DECIPHERD	DRD-healthy	63	0	To describe MND in healthy Chilean individuals
DECIPHERD	DRD-affected	75	0	To describe MND in affected Chilean individuals
DECIPHERD	DRD-pairs	64	32	To describe ∆MND in affected Chilean mother–offspring pairs
22q11.2DS—Chile	22q-CHL	58	0	To describe MND in a known disease
22q11.2DS—Argentina	22q-ARG	26	13	To describe MND and ∆MND in a known disease

DECIPHERD: Decoding Complex Inherited Phenotypes in Rare Disorders Project. DS: Deletion Syndrome. AMR: American. EUR: European. AFR: African.

**Table 2:** Greater effect sizes using OXPHOS genes. Effect sizes were calculated by comparing DRD-healthy to the other cohorts.

		All Mitonuclear Genes			OXPHOS Genes		
	N	Mean	sd	Cohen’s d	Mean	sd	Cohen’s d
DRD-healthy	63	0.6	0.09	—	0.62	0.1	—
DRD-affected	75	0.56	0.12	0.38	0.55	0.13	0.76
22q-CHL	58	0.57	0.13	0.27	0.57	0.13	0.54
22q-ARG	26	0.43	0.24	0.94	0.43	0.25	1.07

DRD: DECIPHERD. CHL: Chile. ARG: Argentina.

## Data Availability

Exome sequencing data for the 22qDS-ARG samples was deposited under bioproject PRJNA1193042. All other datasets used in this study were obtained from previously published work [44,45,46]. Data for plots and the scripts to generate them can be found at https://github.com/berebolledo/results_fondecyt_11220642.

## Supplementary Materials

The following supporting information can be downloaded at: https://www.mdpi.com/article/10.3390/genes16060638/s1, Figure S1: Global ancestry estimation; Figure S2: Correlation between mean MND and European ancestry; Figure S3: Mean MND in cohorts of patients with multiple genetic disorders. Table S1: Haplogroup assignment.

## Abbreviations

The following abbreviations are used in this manuscript:

AFR	African
AMR	Admixed American
ANOVA	Analysis of Variance
CN	Copy Number
DECIPHERD	Decoding Complex Inherited Phenotypes in Rare Disorders
DRD	Decoding Rare Disorders
EUR	European
GWAS	Genome-Wide Association Study
MND	Mitonuclear Discordance
MRT	Mitochondrial Replacement Therapy
NAT	Native American
mtDNA	Mitochondrial DNA
OXPHOS	Oxidative Phosphorylation
SNP	Single-Nucleotide Polymorphism
YBP	Years Before Present
