# Supplementary material for: Exploring the Impact of Mitonuclear Discordance on Disease in Latin American Admixed Populations
Source: Genes (Basel). 2025 May 27;16(6):638. doi: 10.3390/genes16060638 (PMC12192508; doi:10.3390/genes16060638)
Supplement: Supplementary file 1 [file genes-16-00638-s001.zip › Supplementary Figures S1-S3.pdf]

SUPPLEMENTARY MATERIAL

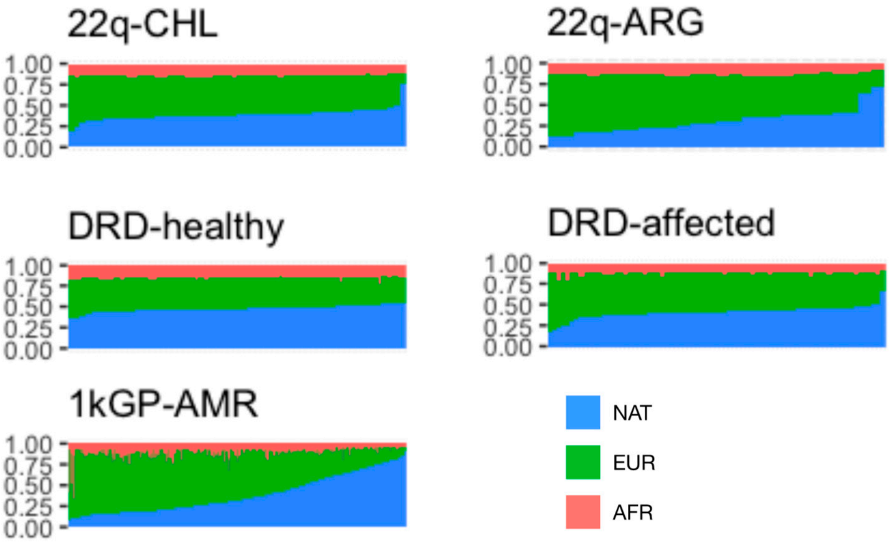

Supplementary Figure S1. Global ancestry estimation. y-axis represents the proportion of each source population. From left to right, samples are sorted in ascending order of Native-American ancestry proportion. NAT: Native American, EUR: European, AFR: African, CHL: Chile, ARG: Argentina, DRD: DECIPHERD (Decoding Complex Inherited Phenotypes in Rare Disorders), 1kGP: 1000 Genomes Project, AMR: Admixed American.

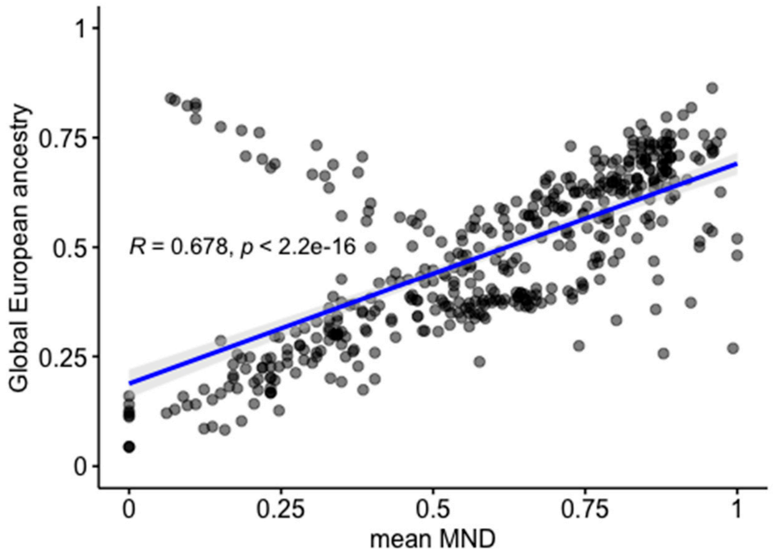

Supplementary Figure S2. Correlation between mean MND and European ancestry.

Scatterplot showing the correlation between mean MND and European ancestry. Linear regression line is shown in blue.

A

B

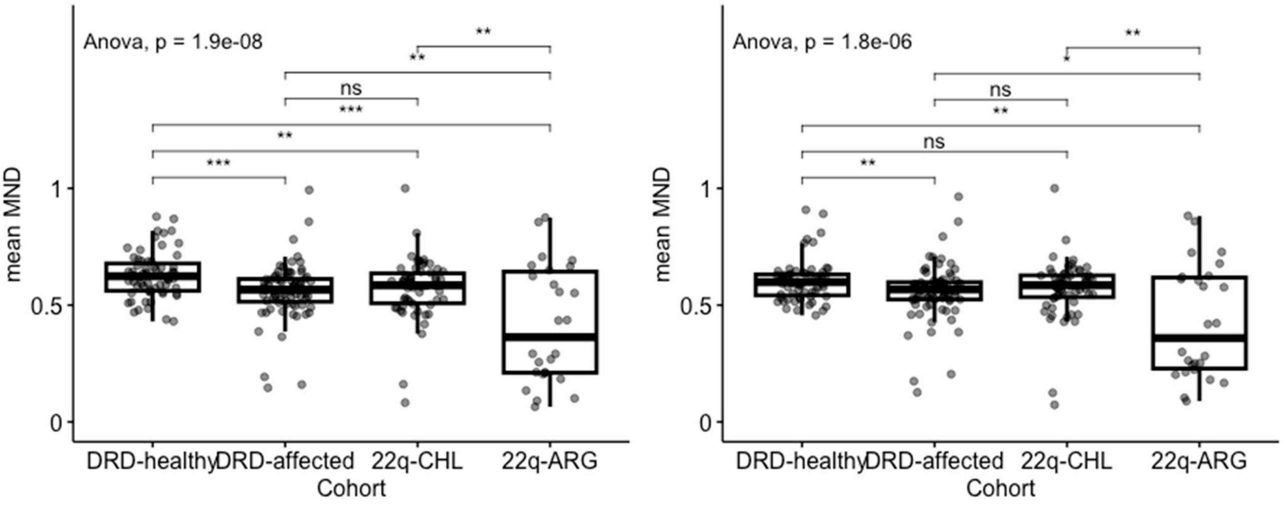

Supplementary Figure S3. Mean MND in cohorts of patients with different genetic disorders. Similarly to figure 2 from the main text, we compared healthy individuals to multiple cohorts of patients. (A) Comparison of mean MDN using high-mt genes. (B) Comparison of mean MDN using low-mt genes.
